# Transmetatarsal Amputation: A Case Series and Review of the Literature

**DOI:** 10.1155/2012/797218

**Published:** 2012-07-03

**Authors:** Ryan McCallum, Mark Tagoe

**Affiliations:** Department of Podiatric Surgery, West Middlesex University Hospital, London TW7 6AF, UK

## Abstract

Foot ulceration is a major cause of morbidity amongst patients with diabetes. In severe cases of ulceration, osteomyelitis and amputation can ensue. A distinct lack of agreement exists on the most appropriate level of amputation in cases of severe foot ulceration/infection to provide predictable healing rates. This paper provides an overview of the transmetatarsal amputation (TMA) as a limb salvage procedure and is written with the perspective and experiences of the Department of Podiatric Surgery at West Middlesex University Hospital (WMUH). We have reflected on the cases of 11 patients (12 feet) and have found the TMA to be an effective procedure in the management of cases of severe forefoot ulceration and infection.

## 1. Introduction

In recent times, increased attention has been placed on the alarming increase in the incidence of diabetes. Diabetic foot ulcers occur in up to 15% of diabetic patients [1], and amputation rates amongst this population have been documented as 11% [2]. In particular cases of severe foot infection, amputation should not necessarily be looked upon as failure of care, but rather the most appropriate intervention for preventing more proximal spread and persistent hospital attendance. Aggressive management of severe foot infection/ulceration can reduce the risk of proximal amputation. 

## 2. Transmetatarsal Amputation 

A proportion of the diabetic community experience serious and debilitating complications associated with their feet, with a 12–25% increased risk of developing foot ulceration [3]. Development of diabetic foot ulceration is often a multifactorial process; however, the presence of influences such as neuropathy and peripheral vascular disease is recognised as significant contributing factor. The neuroischaemic ulceration accounts for 90% of those encountered in the diabetic population [4], and approximately half of diabetic foot wounds develop an infection, the majority involving only soft tissue [5]. In circumstances where soft tissue infection is severe or where underlying bone is infected, amputation may be considered an appropriate line of treatment. Mills et al. [6] recognised that infection and gangrene due to microvascular disease were two major factors that resulted in failure of wound healing, resulting in amputation.

At WMUH, a treatment pathway has been developed for patients with severe foot ulceration/infection who have been deemed suitable candidates for undergoing TMA (see Assessment and Treatment below). Patients are urgently admitted into the hospital and are assessed by the medical and surgical teams, often with input from the tissue viability nurses. The treatment regime is implemented and a significant effort is made to bring the patient on board with the treatment plan. We believe this to be an important factor in improving compliance with the intention of maximising the likelihood of a satisfactory outcome.

**Assessment**

- Medical team assessment and management:
  - stabilisation glycaemic control +/− insulin sliding scale,
  - stabilisation of level of infection via antimicrobial therapy based on clinical presentation and hospital guidelines on diabetic lower limb infection,
  - close monitoring of patient's C-reactive protein, full blood count, temperature, and blood sugar.
- Surgical team assessment:
  - determination of extent of infection,
  - assessment of vascular status,
  - assessment of viable soft tissue.
- Investigations: glycated haemoglobin, C-reactive protein, differential white cell count, culture and sensitivity, doppler, and X-ray.

**Treatment**

- Maintenance of stabilised glycaemic control.
- Decompression of infected tissue:
  - incision and drainage where necessary,
  - deep swabs with culture and sensitivity with appropriate modifications to antibiotic therapy where necessary,
  - negative pressure wound therapy.
- Monitoring of level of infection and determination of healing potential.
- Transmetatarsal amputation with adjunctive soft tissue procedures.
- Orthotist-rocker-bottom shoes with total contact insert.
- Discharge when deemed appropriate.

**Figure 1:**
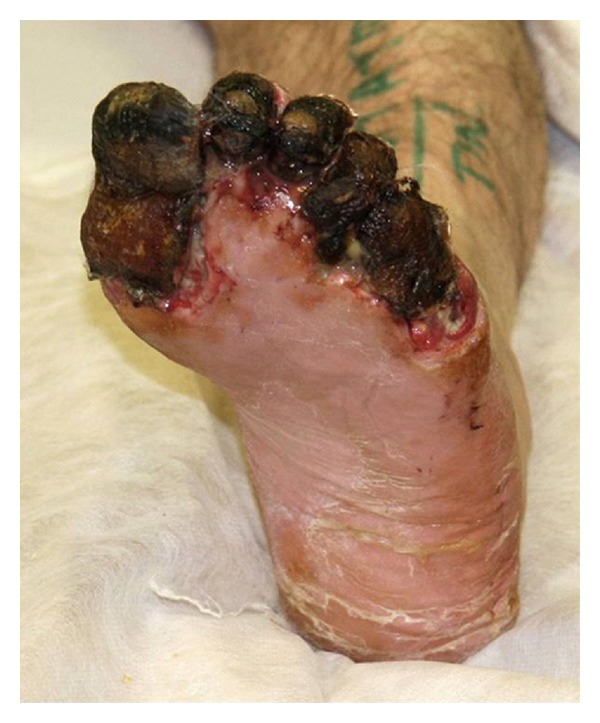


The aim in all cases of diabetic foot infection is to maintain foot function and preserve structure. However, in certain cases, where the soft tissue envelope has been lost or where infection or circulatory impairment has rendered the forefoot nonviable (Figure 1), a transmetatarsal amputation (TMA) might be considered an appropriate option.

A TMA involves removal of the forefoot at the level of the metatarsal shafts with the aim of maximising limb function by maintaining a significant portion of the foot. The procedure was first described by Bernard and Heute [7] for the treatment of trench foot and was later popularised by McKettrick and colleagues [8] as a limb salvaging procedure used for severe diabetic foot complications. The TMA is considered preferable to amputation through the hindfoot or traditional below knee amputation (BKA) and is generally accepted as an effective salvage procedure in cases of forefoot infection, gangrene, and chronic ulceration. The primary advantage is the preservation of a viable weight-bearing platform allowing early ambulation, thus enabling the patients to maintain their independence, whilst maintaining a more acceptable appearance as it may be disguised somewhat with footwear. A partial foot amputation also results in less expenditure of energy during ambulation than more proximal amputations, facilitating mobility and independence [9]. Compared to more proximal amputations, the procedure proves to be the most favourable option with regard to patient satisfaction and function [10].

Table 1 illustrates eleven patients (twelve feet) between June 2006 and December 2011 who have undergone a transmetatarsal amputation under our care. Case J was a nondiabetic case that presented with bilateral forefoot ischaemia as a result of frostbite and underwent bilateral TMA.

All patients remain in hospital until we are satisfied that their recovery is progressing in a satisfactory manner and that domestic circumstances are suitable and appropriate for home discharge. Keeping these high-risk patients in hospital for a longer period immediately postoperatively increases compliance and has, in our experience, lowered readmission rates and further surgery including more proximal amputation.

## 3. Reducing Complications 

Complications are not uncommon following TMA. Anthony et al. [11] reported that 82% of patients who underwent this procedure required further surgery due to postoperative complications, with Pollard and colleagues [12] reporting the need for a more proximal amputation in 32% of cases and hospital mortality (within 30 days of TMA) of 1.98%. These results highlight the need to address factors likely to cause or contribute to subsequent tissue breakdown. 

None of our cohort died within 30-days of their amputation, and only one went on to require a BKA. It must be noted that this is a smaller number of patients in comparison to those previously quoted. This 30 day survival rate betters that of those requiring more proximal amputation with up to 3.6% of BKA patients deceased due to cardiac disease within 30 days [13]. 81% of the patients in this retrospective study had a history of diabetes; however, this was not shown to be a significant predictor of perioperative 30-day mortality. The evidence from the literature illustrates how average survival following major amputation decreases as the level of amputation is sited more proximally. Average survival was noted as 52 months and 20 months for BKA and AKA, respectively. It must be taken into consideration, however, that patients requiring more proximal amputation often have a greater degree of pathology and comorbidities, which would go some way to explaining higher mortality rates.

One of the most significant and well-documented problems with the TMA is the difficulty in predicting successful wound healing. To minimise the likelihood of further tissue breakdown, a number of issues may need to be addressed.

**Figure 2:**
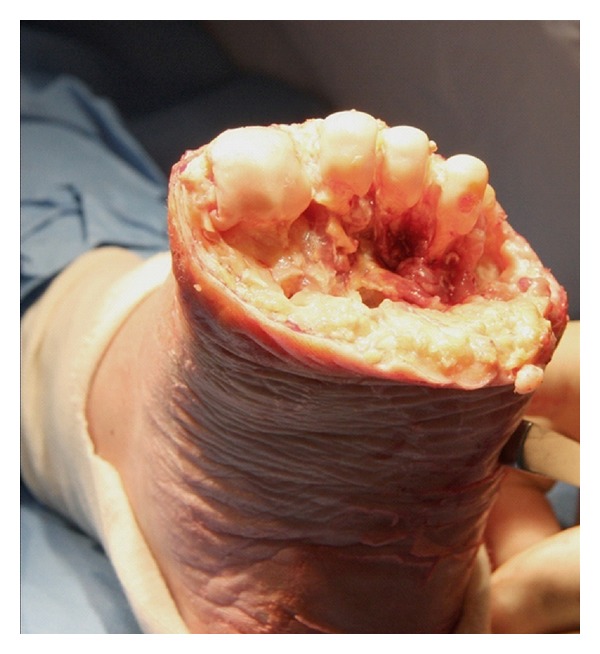


Incision planning is crucial in both providing necessary surgical exposure and also maximising the use of viable soft tissue. A fish-mouth incision is made as distally as possible to maintain as much length to the foot as possible and ensure an adequate plantar flap can be brought dorsally providing soft tissue protection for the metatarsal ends. With the metatarsal heads exposed, clear visualisation of the metatarsal parabola is possible and this allows for the pattern to be maintained when resecting the distal portions of the metatarsals (Figure 2). We aim to maintain the metatarsal parabola in an attempt to prevent peak pressure points on the stump caused by a prominent metatarsal distally. Avascular structures such as tendon stumps and the metatarsophalangeal joint plantar plates are resected as these can pose a nidus for infection.

**Figure 3:**
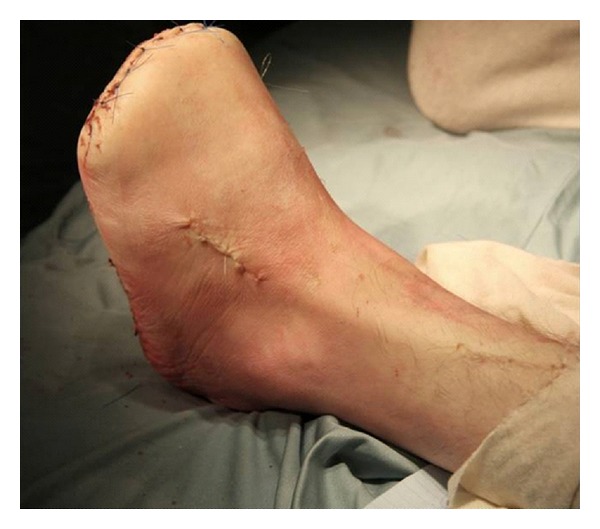
Not the visible tibialis anterior tendon routed laterally following a STATT.

Amputation at the level of the metatarsals causes muscular imbalance with resultant equinovarus deformity due to unopposed action of gastrocnemius, tibialis anterior, and tibialis posterior with the loss of extensor hallucis longus and extensor digitorum longus. This is addressed by performing a gastrocnemius lengthening and a split tibialis anterior tendon transfer (STATT). The STATT involves detachment of the lateral half of the tibialis anterior tendon at its insertion through an initial incision on the dorsomedial aspect of the foot. An incision is made on the anterior aspect of the lower leg and tibialis anterior is identified. The lateral portion of the tendon is passed under the extensor retinaculum to the proximal incision causing a longitudinal split in the tendon. This section of the tendon is then redirected distally and laterally back under the extensor retinaculum to a third incision on the dorsolateral aspect of the foot and is attached to the lateral cuneiform with a bone anchor suture. This allows the foot to sit in a plantigrade fashion in an attempt at reducing peak pressures along the lateral border of the foot (Figure 3). 

 This procedure was routinely carried out except in the case of Patient G. Following delayed healing and a split thickness skin graft at the amputation site, a decision was made not to subject the foot to further surgical insult. We hoped to provide palliative protection in an attempt to prevent further breakdown; however, the patient went onto suffer further ulceration due to subsequent equinovarus deformity. The patient subsequently underwent the aforementioned soft tissue procedures and to date has had no further ulceration or surgery on this foot. 

Tendo Achilles lengthening (TAL) has been shown to effectively reduce peak plantar pressures in the forefoot [14]. La Fontaine et al. [15] alluded to the fact that TAL, although useful, does have its own associated complications such as tendon rupture, heel ulceration, and recurrence of ankle equinus. The senior surgeon in our department (MT) prefers an open gastrocnemius recession, as this procedure is simple to perform with few complications in comparison to the Triple Hoke TAL. Gastrocnemius is well vascularised in comparison with the Achilles tendon and therefore should heal in a more predictable fashion with less chance of tendon rupture. Additionally, where tightness of soleus is not an issue with adequate dorsiflexion at the ankle possible with the knee flexed, lengthening of the Achilles may be seen to unnecessarily weaken the gastrocnemius-soleus complex. Gastrocnemius recession has also been shown to result in superior push-off power with lesser risk of recurrence of equinus in comparison to TAL in cerebral palsy patients [16, 17].

In instances of vascular insufficiency, revascularisation procedures may be required. Predicting the likelihood of successful wound healing depends largely on the patency of the vascular supply and many tests are available to aid in determination of vascular status. Ankle brachial pressure indices (ABPIs) are inexpensive and easy to perform but not necessarily a predictor of healing [18]. Vascular compromise is often masked by calcification of arteries and therefore interpretation of ABPI results should be made with caution. Other physiological tests of wound healing potential such as transcutaneous oxygen pressure and skin perfusion pressure have been reported with encouraging prediction rates following amputation [19, 20]. When significant vascular impairment is encountered, the opinion of the vascular team is sought in the hope that they can improve the patency of blood supply. Revascularisation procedures; however, are not always a viable solution to vascular disease. Patient B underwent a failed popliteal bypass due to the absence of viable arteries in the lower leg and subsequently required a BKA as a result of advanced peripheral vascular disease. In this case, the patient was particularly keen to avoid a knee level amputation and a TMA was agreed upon with significant emphasis placed upon the poor prognosis. In hindsight, a BKA would have been a more appropriate first-line option in view of his ischaemic lower leg. In contrast to this, despite poor prognosis following CT angiogram, Patient D achieved successful wound healing and to date has only had one episode of further ulceration, which required surgical debridement. The amputation site subsequently healed and has remained intact since 5 weeks after this debridement.

The prediction of successful healing and appropriate application of the TMA continue to be based on clinical judgement. In a review of 62 TMAs, Landry et al. were unable to identify any accurate preoperative measures that could predict healing [21]. A significant problem with this patient group is that the majority often have comorbidities that can affect wound healing and therefore predispose to postoperative complications. Landry et al. [22] identified that poor glycaemic control (measured by glycated haemoglobin) is a significant risk factor for progressing to a more proximal amputation. In addition to this, particular risk factors such as diabetes mellitus, infrapopliteal disease, and history of smoking and renal disease can certainly be identified prior to the decision on the most appropriate intervention [23]. The determination of the most appropriate level of amputation remains a vexing surgical problem.

Anthony and colleagues [11] recognised the need for the development of selection criteria to identify those patients who are likely to be best served by a TMA as opposed to a higher level of amputation. 56% of the patients in their study required a more proximal amputation; however, most had significant comorbidities with 89% being graded as American Society Anaesthesiology class 3 or 4. The authors note that the only factor significantly related to more proximal amputation was non-insulin-requiring diabetes. We note a similar trend in our cohort of patients. As yet, no definitive selection criteria for patients undergoing TMA exist.

## 4. Postoperative Considerations 

Consideration of domestic circumstances of individual patients, particularly in situations where patients may have reduced mobility, is crucial in ensuring that patients can be safely discharged from hospital. Following discharge, the influence of healthcare providers is significantly reduced and there is a duty to ensure that an adequate social care framework exists. Ensuring these patients remain in hospital for a longer period postoperatively ensures that wounds are stable upon discharge and should therefore be less likely to breakdown as a result of early noncompliance. Input from physiotherapy to improve ambulation with introduction of walking aids or increasing body strength can be utilised within the community or whilst the patient is an inpatient. The acute stay episode may be used by social services and/or the occupational therapist to assess the patient's home and make amendments as necessary. These simple measures can increase compliance and lower readmission and further surgery rates. WMUH statistics have shown that in the year prior to the implementation of this approach in managing the diabetic foot (2006) 16 BKAs/AKAs were performed. This reduced dramatically with only one BKA/AKA performed in both 2008 and 2009. Undoubtedly, there may be other factors responsible for a reduction in more proximal amputations; however, it is reasonable to infer that this approach can be a contributing factor in such a reduction. 

Pressure reducing techniques such as pressure deflecting dressings, foot orthoses, footwear modifications, and total contact casting may be used because they have good effect in reducing pressure around neuropathic foot ulceration to facilitate wound healing. These modalities may also be used postoperatively following TMA. The clinician must use experience and expertise to determine the most appropriate treatment for each patient on an individual basis. 

Several authors have suggested that patients who have undergone TMA experience minimal functional deficits and that an observer would have difficulty, when a patient is wearing footwear, in telling that a TMA had been performed [10, 24]. However, in a comparison of the functional ability of TMA patients with age-matched controls, Mueller and colleagues [9] found that TMA patients scored much lower in functional tests (some of which involved simple tasks such as simulating eating and putting on a coat) but higher than those with a higher level of amputation in other studies. An obvious explanation for reduced limited function is the decreased foot length. This results in considerable difficulty when performing activities involving transfer of weight onto the forefoot such as walking at normal speed and climbing stairs. Factors such as obesity, visual limitations, and other comorbidities were not taken into consideration in this study; however, poor scores would indicate that these factors are pertinent. The low scoring provides the reader with an insight as to how poor the general well-being of TMA patients can be. 

Diabetic patients with TMA show decreased power at the ankle joint and earlier onset of hip flexor moments and also have limited push-off power therefore relying more on pulling their leg through gait than age-matched controls [25]. Footwear therefore has a role to play in aiding ambulation and improving gait characteristics. As well as enhancing function of the foot, footwear should also protect the residuum and also the contralateral foot from increased loading. An investigation into various footwear modifications for TMA patients showed total contact shoe inserts and rigid rocker bottom soles to both reduce plantar pressures and enhance function. A foot-ankle orthosis and short shoe to match decreased foot length did not enhance functional stability, and these were poorly tolerated by patients [26]. Our patients are routinely referred on an urgent basis to the orthotist within our hospital for the provision of bespoke footwear with a rocker soled shoe and total contact insert.

## 5. Conclusion 

Transmetatarsal amputation is an effective procedure in the treatment of severe forefoot infection/ulceration. Where the forefoot is rendered nonviable, the patient can return to full ambulation and independence providing postoperative complications are avoided or managed appropriately. The TMA does not come without risk, and high failure rates have been well documented throughout the literature. Consideration of the adjunctive soft tissue procedures and mechanical post-operative modalities available is important in providing the greatest chance of avoiding further breakdown. This highlights the need for careful patient selection and also recruitment of the whole multidisciplinary team. The benefit of reduced morbidity and maintenance of function when successful make the procedure preferable to more proximal amputations in our experience.

## Figures and Tables

**Table 1:** 

Patient	Age/sex at time of TMA	Diabetes	Date of TMA	Adjunct procedures	Current status
A	62/M	Type II	05/06/06	STATT, GR	Healed
B	40/M	Type II	04/08/06	Popliteal bypass, BKA	Deceased
C	57/M	Type I	23/11/07	STATT, GR	Healed
D	64/M	Type II	08/09/08	I&D	Healed
E	50/M	Type II	18/10/08	Pan talar fusion scheduled	Deceased
F	47/M	Type I	02/02/09	STATT, GR	Healed
G	46/M	Type I	07/05/07	Skin graft, I&D, STATT, GR	Healed
H	56/F	Type I	16/03/09	STATT, GR	Healed
I	51/M	Type I	27/04/09	STATT, GR, I&D	Healed
J	46/M	Nondiabetic	11/02/10	STATT, GR	Healed
K	46/F	Type II	16/12/11	STATT, GR	Healed

****STATT: split tibialis anterior tendon transfer; GR: gastrocnemius recession; BKA: below knee amputation; I&D: incision and drainage.
